# Characterisation of a live *Salmonella* vaccine stably expressing the *Mycobacterium tuberculosis* Ag85B–ESAT6 fusion protein

**DOI:** 10.1016/j.vaccine.2009.09.007

**Published:** 2009-11-16

**Authors:** Lindsay J. Hall, Simon Clare, Derek Pickard, Simon O. Clark, Dominic L.F. Kelly, Moataz Abd El Ghany, Christine Hale, Jes Dietrich, Peter Andersen, Philip D. Marsh, Gordon Dougan

**Affiliations:** a Wellcome Trust Sanger Institute, Wellcome Trust Genome Campus, Hinxton, Cambridgeshire CB10 1SA, UK; b Centre for Emergency Preparedness and Response, Health Protection Agency, Porton Down, Salisbury, Wiltshire SP4 0JG, UK; c Department of Infectious Disease Immunology, Statens Serum Institute, Artillerivej 5, DK-2300 Copenhagen, Denmark

**Keywords:** *Salmonella* vector, Mucosal vaccine, *M. tuberculosis*

## Abstract

A recombinant *Salmonella enterica* serovar Typhimurium (*S.* Typhimurium) vaccine strain was constructed that stably expressed the *Mycobacterium tuberculosis* fusion antigen Ag85B–ESAT6 from the chromosome. Live oral vaccination of mice with the *Salmonella*/Ag85B–ESAT6 strain generated a potent anti-Ag85B–ESAT6 T_H_1 response with high antibody titres with a IgG2a-bias and significant IFN-γ production lasting over a 120-day period. When mice primed with the *Salmonella*/Ag85B–ESAT6 vaccine were mucosally boosted with the Ag85B–ESAT6 antigen and adjuvant the IFN-γ responses increased markedly. To determine the protective efficacy of this vaccine strain, guinea pigs were immunised and followed for a 30-week period after aerosol challenge with *M. tuberculosis*. The heterologous prime-boost strategy of live *Salmonella* vaccine followed by a systemic boost of antigen and adjuvant reduced the levels of *M. tuberculosis* bacteria in the lungs and spleen to the same extent as BCG. Additionally, this vaccination regimen was observed to be statistically equivalent in terms of protection to immunisation with BCG. Thus, live oral priming with the recombinant *Salmonella*/Ag85B–ESAT6 and boosting with Ag85B–ESAT6 plus the adjuvant LTK63 represents an effective mucosal vaccination regimen.

## 1 Introduction

Currently, nearly two billion people are infected with *Mycobacterium tuberculosis*. Annually, nine million people become ill with *M. tuberculosis*-associated tuberculosis and approximately two million die from the disease worldwide, mostly in developing countries. Tuberculosis is also a leading cause of death among Human Immunodeficiency Virus (HIV) infected people, with co-infection accounting for up to a third of deaths associated with Acquired Immunodeficiency Syndrome (AIDS), worldwide [1]. Along with HIV/AIDS and malaria, tuberculosis remains as one of the three main killers among global infectious diseases [2]. Currently the only licensed vaccine in the fight against tuberculosis is Bacille Calmette-Guérin (BCG). This live attenuated vaccine has been in use for over 80 years and has displayed up to 80% efficacy against serious forms of the disease, e.g., meningitis, in children. However, efficacy in adults against pulmonary tuberculosis ranges from 0 to 80% in different trials [3].

*M. tuberculosis* is a pathogen which normally infects individuals via the mucosal tissue of the respiratory tract following inhalation of infectious droplets. Many new tuberculosis vaccination strategies have concentrated on the parenteral route of administration, which is also how the current live BCG vaccine is delivered in most parts of the world. However, a number of studies have shown that delivery via mucosal routes may elicit a local respiratory mucosal immunity which may increase protection against *M. tuberculosis* infection [4–7]. Different strategies can be used to deliver vaccine antigens via the mucosal route, with living attenuated bacterial vaccines being among the most promising candidates. The attenuated bacterial antigen vectors can be used to induce immunity to their corresponding pathogenic strain or they can be modified to deliver protective heterologous (foreign) antigens, plasmid DNA or other macromolecules such as immune modulators [8]. Attenuated derivatives of *Salmonella enterica* have been proposed as vehicles for the mucosal delivery of heterologous antigens and as a basis for multivalent vaccines. In fact, strains of *S.* Typhi and *S.* Typhimurium were among the first bacterial recombinant vaccine vectors used to deliver heterologous antigens [9,10]. Oral vaccination with live attenuated *Salmonella* vectors can result in the generation of both *Salmonella* and heterologous antigen specific humoral and cellular immune responses, normally biased towards T_H_1 [11,12]. Considerable progress has been made in clinical studies with attenuated *S.* Typhi-based vaccines, which can be used both as a more effective typhoid vaccine and for delivery of heterologous antigens [13–15]. Since humans are the only known natural host for *S.* Typhi, many strategies for producing attenuated vaccine vectors are initially assessed using *S.* Typhimurium [16–20].

Two of the major antigens produced by *M. tuberculosis* during infections are antigen 85B (Ag85B), a 31 kDa mycolyl transferase involved in cell wall biogenesis, and early secreted antigenic target-6 (ESAT6), a small 6 kDa protein possibly involved in immune modulation [21–24]. Ag85B and ESAT6 are both capable of inducing strong immune responses in a number of animal models [25–28]. Previous work has shown that the fusion of Ag85B–ESAT6 is more immunogenic, and gives higher levels of protection compared to the individual antigens when administered parenterally [29–32]. In addition, intranasal (i.n.) immunisation regimens with Ag85B–ESAT6, both with the mutant heat labile toxin (LT) adjuvant LTK63 or a derivative of cholera toxin CTA1-DD/ISCOMs, have shown promise [33,34]. Both studies demonstrated potent anti-Ag85B–ESAT6 immune responses, in addition to significant protection after *M. tuberculosis* challenge in a murine model [4,5]. In this present study we evaluated the immunogenicity and protective efficacy of a novel recombinant *S.* Typhimurium vaccine expressing the Ag85B–ESAT6 fusion antigen as part of a mucosal prime/boosting vaccination regimen with Ag85B–ESAT6 protein and LTK63 adjuvant.

## 2 Materials and methods

### 2.1 Bacterial strains, primers and plasmids

*S.* Typhimurium SL3261, which harbours an attenuating mutation in the *aroA* gene, was used as the base vector for all live vaccine studies [35] and was routinely grown in (Luria–Bertani) LB-broth supplemented with l-phenylalanine, l-tryptophan, l-tyrosine (40 μg/mL each) along with p-aminobenzoic acid and 2,3-dihydroxybenzoic acid (10 μg/mL).

For the isolation of the expression cassette containing the Ag85B–ESAT6 fusion under the control of the *lacZ* promoter, plasmid pMCT6 was obtained from the Statens Serum Institute, Denmark [29,36]. PCR fragments generated from this fusion were initially ligated into pGEM-Teasy (Invitrogen) for easier manipulation. Plasmid p2795, required for integration of the expression cassette into the *phoN* gene of the SL3261 genome, was a kind gift of Michael Hensel [37]. The red recombinase plasmid, pKD46 was utilised for homologous recombination of the expression cassette into the *phoN* region of SL3261 chromosome [38]. Primers were designed using MacVector software and are shown in Table 1. When required, kanamycin (Invitrogen) was used at 50 μg/mL and ampicillin (Roche) at 100 μg/mL.

### 2.2 Construction of recombinant *Salmonella* SL3261mycolacZ

**Fig. 1:**
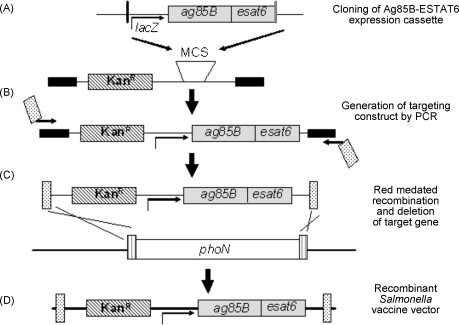
Schematic diagram for the construction of targeting construct and chromosomal integration of expression cassette. (A) Expression cassette consists of a constitutive promoter (*lacZ*), a gene fragment encoding the model vaccine fusion antigen Ag85B–ESAT6 (grey symbol). Expression cassette for expression of Ag85B–ESAT6 was inserted into the multiple cloning site of p2795 within SalI (black line) and BamH1 (grey line) sites. p2795 contains the kanamycin resistance gene (diagonal lines) and binding sites for primers (black symbol). (B) The targeting construct consisting of expression cassette and resistance gene was amplified by knock-in primers containing sequences complementary to the chromosomal target gene, *phoN* (dots). (C) *S.* Typhimurium strain SL3261 harbouring pKD46 for expression of Red recombinase was transformed with linear DNA of targeting construct. (D) Recombinant clones with replacements of a chromosomal target gene, *phoN* (white symbol) by the targeting construct were selected. Modified from [37].

The red recombinase technique devised by Datsenko and Wanner was utilised for integration of the expression cassette into the *phoN* gene of the SL3261 genome [38]. This required the use of plasmid p2795 which was specifically designed by Husseiny and Hensel for integration of expression cassettes into the genomes of bacteria using the red recombinase system [37]. This vector permitted mobilisation of the Ag85B–ESAT6 fusion expressed under the *lacZ* promoter into the *phoN* site of *S.* Typhimurium SL3261. The construction of the integrated cassette is illustrated in Fig. 1 and is briefly described below. We cloned the expression cassette into the multiple cloning site (MCS) of p2795, which contains the kanamycin resistance gene for selection purposes, using enzymes BamHI and SalI (Fig. 1A). Using primers phoNhensF and phoNhensR, we amplified the entire region including *phoN* sequences at each end, the kanamycin gene and lacZ-Ag85B–ESAT6 cassette (Table 1 and Fig. 1B). The resulting PCR product was treated with DpnI to remove any plasmid DNA template.

For integration into the *phoN* region of SL3261 chromosome, the PCR product was electroporated into SL3261 harbouring the red recombinase plasmid, pKD46 (Fig. 1C). 2 μg of PCR product was used for this procedure. Kanamycin resistant colonies were selected and were further plated at 37 °C so as to encourage loss of the pKD46 plasmid. The chromosome bearing the Ag85B–ESAT6 expression cassette was then mobilised into a new *S.* Typhimurium SL3261 derivative via transduction using the bacteriophage P22 [39]. Remaining kanamycin resistant colonies were retained and Western blots performed to confirm expression of the Ag85B–ESATB fusion proteins via the *lacZ* constitutive promoter. Primers phoNseqF, phoNseqR and mycoseqR were used to confirm sequence. One isolate was named SL3261mycolacZ and was used in all further immunisation experiments (Table 1 and Fig. 1D).

### 2.3 Analysis of Ag85B–ESAT6 expression

In order to determine expression levels of Ag85B–ESAT6 from SL3261mycolacZ, LB-broth cultures were harvested by centrifugation and resuspended in phosphate-buffered saline (PBS) (pH 7.4) to an optical density at 600 nm (OD_600_) of 1. The suspension was then mixed with an equal volume of 2× sodium dodecyl sulphate (SDS) loading buffer and separated by SDS-polyacrylamide gel (SDS-PAGE) electrophoresis using a 4–20%-gradient gel (Bio-Rad). Proteins were transferred to polyvinylidene fluoride membranes (Immobilon-P) by Western transfer and detected with monoclonal anti-Ag85B antibody (1:50) obtained from the Statens Serum Institute, Denmark.

### 2.4 Vaccination of mice with serovar Typhimurium

For inoculation of mice, the serotype Typhimurium strain was grown statically overnight in LB-broth supplemented with kanamycin as appropriate. Bacteria were harvested by centrifugation, washed, and then suspended in PBS (pH 7.4) to approximately 5 × 10^9^ CFU mL^−1^. Serial dilutions of the bacterial culture were also spotted onto LB agar plates and cultured at 37 °C overnight to confirm concentration. Groups of 6–8-week-old female C57BL/6 mice (obtained from Harlan Ltd., UK, Bicester) were inoculated orally by gavage with 200 μL of bacterial suspension each. On days 21, 50, 57 and 120 blood was collected and sera prepared by standard techniques and stored at −20 °C for further analysis. In addition, on days 50, 57 and 120, groups of 5 animals were euthanized to harvest splenocytes and lung and nasal lavages performed.

### 2.5 Vaccination of mice using the prime-boost regimen

Mice were primed orally with SL316mycolacZ on day 0 and intranasally (i.n.) boosted on day 50 with either PBS or 20 μg LTK63 (negative controls) or 20 μg LTK63 plus 25 μg Ag85B–ESAT6 in a volume of 15–20 μL/nostril. As a positive control, one group of mice were i.n. immunised with 1 μg LT and 25 μg Ag85B–ESAT6 on day 0 as this has previously been shown to induce both antibody and cytokine responses (unpublished observations). The adjuvants LT and LTK63 were a kind gift from Novartis, Italy, and purified Ag85B–ESAT6 was obtained from the Statens Serum Institute, Denmark. Proteins were mixed immediately before use. Blood samples were obtained on day 57 and day 120. Groups of 5 animals were euthanized at the same time-points to harvest splenocytes and perform lung and nasal lavages.

### 2.6 Evaluation of antibody responses

Serum samples from mice post-immunisation were analysed for the presence of total Ig, IgG, IgG1, IgG2a or IgA. Briefly, ELISA plates (Nunc Maxisorp) were coated overnight at 4 °C with 50 μL of a 2 μg/mL solution of purified Ag85B–ESAT6 in coating buffer (0.1 M Na_2_HPO_4_ at a pH of 9) and then blocked with 3.0% BSA at room temperature for 1 h. Serum samples were diluted in 0.1% BSA starting at 1:100. Each plate contained control wells with preimmune sera, PBS (pH 7.4), or a known positive immune serum and incubated for 1 h at 37 °C. Antibodies conjugated to HRP were diluted 1:1000 and plates incubated for a further 30 min at 37 °C. The level of HRP associated with each well was then determined using Sigma Fast *o*-phenylenediamine dihydrochloride (50 μL per well) colorimetric substrate. The reaction was stopped after 15 min by the addition of 2.5 M H_2_SO_4_. The OD_490_ was determined, and the titre was expressed as the reciprocal of the dilution giving an OD of 0.3.

Lung and nasal lavages, performed post-mortem, were used to measure mucosal antibody responses. Lavages were performed using 1 mL of ice-cold PBS containing a cocktail of protease inhibitors (Roche), which was flushed in and out of the lungs and nasal passages with a fine-tipped Pasteur pipette inserted via the trachea. Lavage fluids were then stored at −20 °C prior to analysis utilising the above ELISA protocol modified as follows. Lavage fluid was diluted in 0.1% BSA starting at 1:10. A rat monoclonal anti-mouse IgA, conjugated to biotin (BD Biosciences), was used as the secondary antibody at 1:1000. To detect the biotin-conjugated antibody, plates were washed four times in PBS-T and then 50 μL of streptavidin-HRP diluted at 1:1000 in PBS-T was added to each well. Plates were developed and titres measured as described above.

### 2.7 Cytometric bead array cytokine analysis

Mice were sacrificed and their spleens removed and seeded, in duplicate at a concentration of 5 × 10^5^ cells/well. Splenocytes were stimulated with either Ag85B–ESAT6 or Con A (both at 5 μg/mL) or RPMI medium. Plates were incubated at 37 °C and 5% CO_2_ for 36–48 h. Cytokines IL-2, IL-4, IL-5, IL-6 and IFN-γ were analysed using cytometric bead analysis flexi-kits (BD Biosciences) and assays were performed per the manufacturer's instructions. Samples were then analysed on a FACSAria flow cytometer (BD Biosciences). The limit of detection was ∼8 pg/mL for all cytokines.

### 2.8 Guinea pig challenge study

Protection was evaluated in outbred female Dunkin–Hartley guinea pigs (weighing between 250 and 300 g), obtained from Harlan Ltd., UK, Bicester. Vaccine efficacy was evaluated in groups of eight guinea pigs in comparison to positive controls that received 5 × 10^4^ CFU of BCG Danish strain 1331 and PBS negative controls delivered subcutaneously (s.c.). SL3261mycolacZ was delivered orally in 1 mL of suspension (total dose of 5.5 × 10^8^ CFU) and delivered at the back of the mouth slowly to ensure the full dose was swallowed. Four weeks later, one group of animals was boosted with 20 μg Ag85B–ESAT6 plus 50 μg of LTK63 delivered s.c. in a total volume of 250 μL.

Animals were rested for 10 weeks post-vaccination before being challenged with an aerosol dose of *M. tuberculosis*. Guinea pigs were aerosol challenged using a contained Henderson apparatus as previously described [40,41]. Fine particle aerosols of *M. tuberculosis* H37Rv, with a mean diameter of 2 μm (diameter range, 0.5–7 μm) [42], were generated using a 3-jet collision nebuliser and delivered directly to the snout of each animal. The aerosol was generated from a water suspension containing 5 × 10^6^ CFU mL^−1^ in order to obtain an estimated retained, inhaled dose of 20–50 CFU/lung [41]. The intention was to infect the animals with a relatively low dose aerosol challenge since this is within the range of doses conventionally used in guinea pig models of tuberculosis. Following aerosol challenge, the guinea pigs were housed at ACDP containment level 3 and monitored regularly for weight changes as an indicator of disease. The animals were culled humanely at study end (30 weeks post-challenge) or at the humane end-point defined as 20% loss of maximal body weight with peritoneal overdoses of sodium pentobarbitone.

When a guinea pig was euthanized, the spleen and lungs were aseptically removed and stored at −20 °C until processed for bacteriology. Frozen tissues were thawed and homogenised and viable counts were performed on Middlebrook 7H11 + OADC selective agar. Plates were incubated at 37 °C for 4 weeks before counting the number of *M. tuberculosis* CFU. Vaccine efficacy was assessed in terms of reduction in bacterial counts in lung and spleen. Guinea pig experimental work was conducted according to UK laws for animal experimentation and was approved by a local ethical committee at the Health Protection Agency, CEPR, Porton Down, UK.

### 2.9 Statistical analysis of data

Experimental results were plotted and analysed for statistical significance utilising Prism4 software (GraphPad Software Inc.).

## 3 Results

### 3.1 Construction of recombinant *Salmonella* vaccine and *in vitro* expression of antigen Ag85B–ESAT6

Several genetic approaches have been developed that facilitate the chromosomal expression of heterologous antigens in *Salmonella* and one such system, based upon *phoN* as an integration site, was selected for this study [37]. This approach relies on the exploitation of a single copy of the heterologous gene to drive immunologically relevant levels of antigen expression. Chromosomal integration has the advantage of providing a stable platform for antigen expression, favouring the subsequent exploitation of the technology in applied clinical studies. Here, the *Escherichia coli* promoter *lacZ* was chosen to drive constitutive expression of the Ag85B–ESAT6 protein. Previously, both the *ssaG* promoter from *Salmonella* pathogenicity island-2 (SPI-2) and the *nirB* promoter from *E. coli* were used to drive *in vivo* expression of Ag85B–ESAT6 from plasmids. However, using multi-copy plasmids and three doses of the recombinant *Salmonella* strains, we found that antigen expression and subsequent immune responses generated in immunised mice were significantly lower than those seen using a single dose of *Salmonella* harbouring the chromosomal driven antigen expression from the constitutive *lacZ* promoter (Hall et al., unpublished). This is similar to what was reported by Husseiny et al. when they compared chromosomal integration of an *in vivo* inducible (*sseA* from SPI-2) and a constitutive promoter to drive antigen expression in a *Salmonella* vaccine vector [37].

**Fig. 2:**
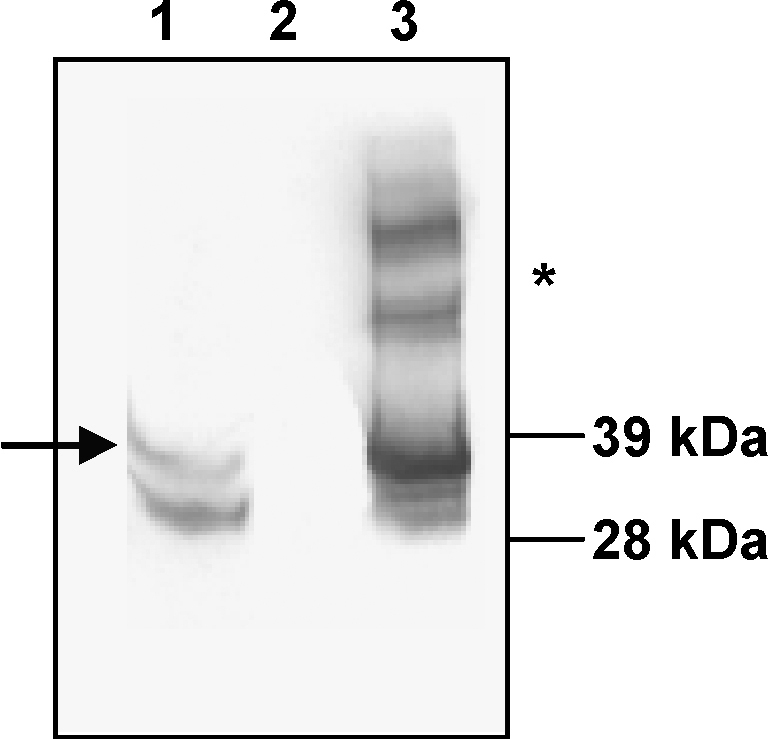
Ag85B–ESAT6 expression by recombinant *S.* Typhimurium vaccine strain. Bacterial lysates, were separated by SDS-PAGE electrophoresis (4–20% gradient gel; 2 × 10^7^ bacteria per lane), blotted onto nitrocellulose, and probed with a monoclonal antibody against native Ag85B. Lanes 1 and 2 shows log phase static cultures of SL3261mycolacZ and the SL3261 parental strain, respectively. The protein was identified by comparison to 1 μg of purified recombinant Ag85B–ESAT6 standard (Lane 3). The protein band corresponding to Ag85B–ESAT6 is indicated by the arrow. The * indicates dimers of Ag85B–ESAT6 in the purified protein.

The red recombinase approach was utilised for integration of the expression cassettes into the chromosome of *S.* Typhimurium SL3261 (Fig. 1). Whole-cell lysates of SL3261mycolacZ, or of the parental control strain, were grown statically at 37 °C to allow Ag85B–ESAT6 expression and these were then probed with a monoclonal antibody against Ag85B. A positive control of purified Ag85B–ESAT6 protein was also probed. The Western blots analysis identified two major immuno-reactive polypeptides in both SL3261mycolacZ lysates and with purified Ag85B–ESAT6 protein. These were absent in similar SL3261 lysates. The electrophoretic mobility of these bands corresponded to that expected for the Ag85B–ESAT6 fusion protein, 37 kDa and the single Ag85B protein from breakdown of the fusion protein at 31 kDa (Fig. 2). To test stability *in vivo*, mice were orally immunised with the SL3261mycolacZ strain as described in Section 2. Plating on selective agar with or without kanamycin at 7 days following challenge indicated that the *S.* Typhimurium construct was stable.

### 3.2 Humoral immunogenicity of recombinant *Salmonella* vaccine in an oral priming i.n. protein boost regimen

#### 3.2.1 Total Ig serum anti-Ag85B–ESAT6 response

**Fig. 3:**
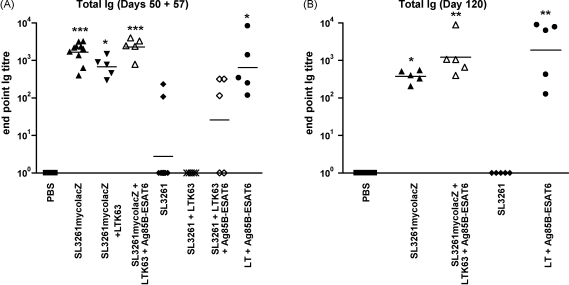
Serum Ig anti-Ag85B–ESAT6 antibody response in C57BL/6 mice following oral *Salmonella* prime and i.n. protein boost. Mice were orally vaccinated with approximately 5 × 10^9^ CFU of SL3261mycolacZ on day 0 and then boosted with 25 μg Ag85B–ESAT6 + 20 μg LTK63 or appropriate antigen controls on day 50. Negative control mice were immunised with wild-type SL3261 or PBS, and then also boosted on day 50 with suitable antigens. Positive controls received 1 μg LT plus 10 μg Ag85B–ESAT6 i.n. at day 0. Mice were left for 21, 50, 57 and 120 days and then sample bled to determine anti-Ag85B–ESAT6-specific Ig antibodies, which were determined by ELISA. (A) Graphs express total antibody titres using a cut off of OD 0.3 on days 50 (prior to boost) and 57 (plus boost) and on (B) day 120. The black bar shows the geometric mean from the group with the significant values of *p < 0.05; **p < 0.01 and ***p < 0.001 as determined using the Kruskal–Wallis test followed by Dunn's Multiple Comparison test compared to negative controls.

In order to determine if SL3261mycolacZ was effective in stimulating humoral immune responses, sera collected from mice at various time-points after oral immunisation were tested to ascertain the kinetics of anti-Ag85B–ESAT6 Ig antibody responses by ELISA. C57BL/6 mice were orally immunised with either, SL3261mycolacZ, SL3261 or PBS as described in Section 2. Mice were then boosted i.n. with PBS, LTK63 or LTK63 and Ag85B–ESAT6 at day 50. The antibody response that followed a single immunisation with SL3261mycolacZ administered orally was found to be significant (*p* < 0.001), 21 days post-priming, when compared to PBS and SL3261 vaccinated mice (data not shown). These anti-Ag85B–ESAT6 titres were observed to increase over 10-fold by day 50, where 100% of the mice showed significant (*p* < 0.001) titres (Fig. 3A). Indeed, 6 months (day 120) after initial immunisation with SL3261mycolacZ, mice were still observed to have significantly robust (*p* < 0.05) Ig titres (Fig. 3B). Boosting i.n. with LTK63 and Ag85B–ESAT6 in SL3261mycolacZ-primed mice appeared to enhance serum Ig anti-Ag85B–ESAT6 responses at both 7 days and 70 days post-boost (Fig. 3A and B, respectively). Mice vaccinated with SL3261mycolacZ, and then boosted with either PBS or LTK63, did not show any increase in Ag85B–ESAT6-specific titres 7 days after boosting (Fig. 3A). As expected, mice primed with SL3261 with/without a boost of either PBS or LTK63 did not have any significant (*p* > 0.05) anti-Ag85B–ESAT6 titres (Fig. 3A). Mice orally primed with SL3261 and i.n. boosted with purified Ag85B–ESAT6 and LTK63 did have modest anti-Ag85B–ESAT6 titres 7 days after boosting, however only 60% of the animals sero-converted (Fig. 3A). As expected, the positive control group, i.e. those animals i.n. immunised with LT and Ag85B–ESAT6, attained high titres throughout the study (Fig. 3).

#### 3.2.2 IgG sub-class responses

**Fig. 4:**
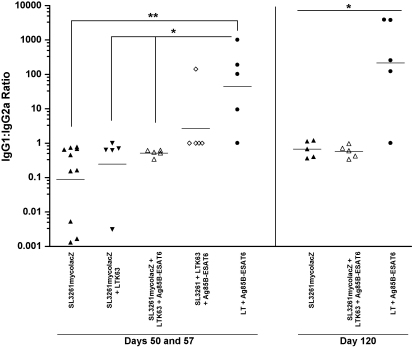
Ag85B–ESAT6-specific IgG1:IgG2a antibody titre ratios. C57BL/6 mice were immunised as described in Fig. 3 legend. Negative control mice, i.e. PBS and SL3261 immunised had no detectable IgG subtype titres (data not shown). Titres shown were determined in individual serum samples obtained on days 50, 57 and 120 after immunisation. Bars represent geometric means. Statistical significance was determined by using the Kruskal–Wallis test followed by Dunn's Multiple Comparison test (*p < 0.05 and **p < 0.01).

Sera collected on days 50, 57 and 120 from primed and/or boosted mice were further analysed to ascertain Ag85B–ESAT6-specific IgG_1_:IgG_2a_ sub-class ratios as an indirect assessment of the T-helper cell response bias (Fig. 4). On all days, mice primed with SL3261mycolacZ with/without LTK63 or Ag85B–ESAT6 plus LTK63 boost were found to be more IgG2a-biased, as depicted by the low IgG1:IgG2a ratios. However, there were no IgG_1_:IgG_2a_ profiles that were statistically different between these experimental groups. Mice vaccinated with the SL3261 parental strain and then boosted with adjuvant and Ag85B–ESAT6 had an IgG1 biased response; however, it was not statistically different from those animals immunised with the recombinant vaccine strain, SL3261mycolacZ, plus or minus a boost. Importantly, mice dosed once with the adjuvant LT and Ag85B–ESAT6 engendered a significantly greater (*p* < 0.05) proportion of IgG_1_-specific anti-Ag85B–ESAT6 antibodies than those primed with SL3261mycolacZ and consequently boosted over all time-points tested.

#### 3.2.3 Serum and mucosal IgA response

**Fig. 5:**
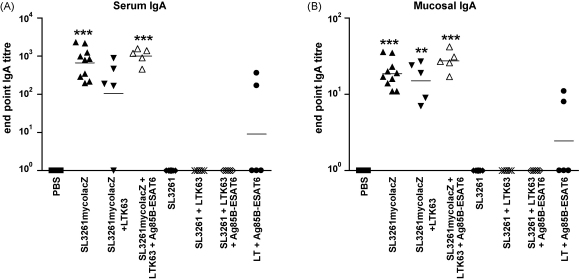
Systemic and mucosal IgA induced by immunisation with *Salmonella* live vector vaccine. Antibody titres were measured following oral prime immunisation with SL3261mycolacZ vaccine candidate and i.n. boosting with antigen plus adjuvant. (A) Graph shows titres obtained from individual serum samples collected on days 50 (prior to boost) and then on days 57 (plus boost). (B) Lung and nasal wash IgA titres are also shown. Serum and washes isolated from mice immunised with SL3261 parental strain or PBS served as negative controls. For group and statistics details see legend of Fig. 3.

The systemic and mucosal IgA response following administration of the SL3261mycolacZ inoculum was also analysed. The levels of anti-Ag85B–ESAT6 IgA in the serum of immunised mice is shown in Fig. 5A. The anti-Ag85B–ESAT6 IgA titres were significantly higher (*p* < 0.001) in mice primed with SL3261mycolacZ with or without boosting when compared to negative control animals, i.e. those immunised with PBS or SL3261. However, only 80% of the mice that received SL3261mycolacZ plus an LTK63 boost sero-converted and were found not to be significantly different from the negative control mice. The anti-Ag85B–ESAT6 IgA response in those mice i.n. immunised with LT and Ag85B–ESAT6 was 100-fold lower than that elicited by the other groups immunised with the candidate vaccine and only 40% of animals sero-converted. At the final time-point (day 120), the titres of anti-Ag85B–ESAT6 IgA induced by immunisation with SL3261mycolacZ returned to levels similar to the negative control mice (i.e. undetectable). Notably, only one mouse orally immunised with SL3261mycolacZ and i.n. boosted with Ag85B–ESAT6 antigen and LTK63 still had detectable IgA levels at this time-point (data not shown).

Since vaccination through the oral and nasal route has the potential to induce mucosal protective responses, we investigated the induction of IgA in nasal and lung washes of mice immunised with SL3261mycolacZ vaccine. We also compared the levels of mucosal IgA after the i.n. boost. Mucosal Ag85B–ESAT6-specific IgA titres were demonstrated in both nasal and lung washes following immunisation with SL3261mycolacZ; significant responses (*p* < 0.01) were readily detected 50 days after the first dose and were still present 7 days after boosting (day 57) (Fig. 5B). However, mucosal IgA could not be detected in any groups after completion of the experiment at day 120 (data not shown). A slightly higher titre of IgA was observed in lung and nasal washes from mice immunised with SL3261mycolacZ and boosted i.n. with Ag85B–ESAT6 antigen plus LTK63 adjuvant than in mucosal washes from those receiving SL3261mycolacZ with or without the negative control boosts. IgA antibodies were undetectable in mice immunised with SL3261 or PBS (negative controls) including those mice receiving SL3261 plus the LTK63 and Ag85B–ESAT6 boost.

### 3.3 Evaluation of cytokine responses after recombinant *Salmonella* vaccination

**Fig. 6:**
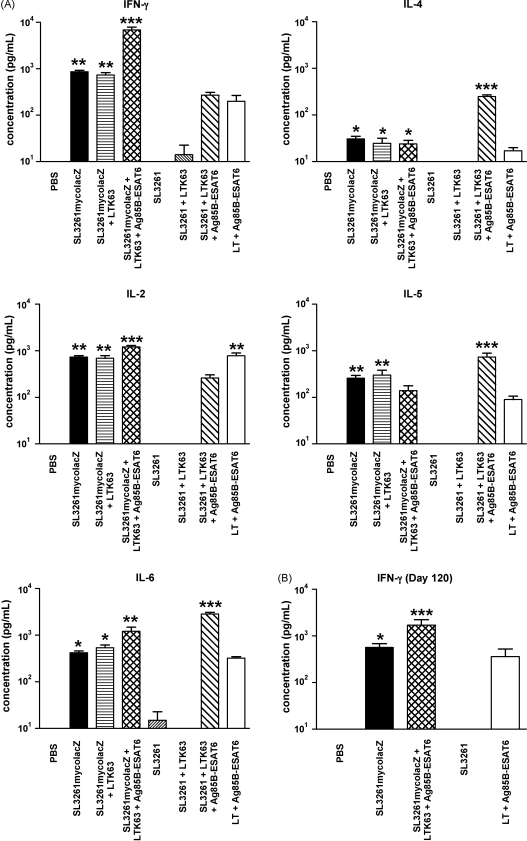
Ag85B–ESAT6-specific cytokine responses in C57BL/6 mice primed with *Salmonella* live vector vaccine and boosted with LTK63 and Ag85B–ESAT6. Ag85B–ESAT6-specific cytokine secretion of splenocytes from mice primed by oral immunisation with SL3261mycolacZ, SL3261, or PBS (day 0) and boosted by i.n. administration of Ag85B–ESAT6 plus LTK63 or appropriate controls (day 50) are shown. Spleens were harvested on days 50 and 57, and 120. (A) Figure shows cytokine levels from stimulated splenocytes on days 50 and 57. Cytokine responses were measured upon *in vitro* stimulation with Ag85B–ESAT6 for 36–42 h including IFN-γ, IL-4, IL-2, IL-5 and, IL-6. (B) IFN-γ levels from splenocytes isolated on day 120 are also shown. Cells were also stimulated with ConA (positive control) and RPMI^+^ media (negative control). Columns represent the mean (±SD) stimulation indices of splenocytes from 5 to 10 animals per group. The increase in cytokine levels between SL3261mycolacZ-primed, i.n.-boosted mice, compared to negative control mice, is indicated (*p < 0.05; **p < 0.01; ***p < 0.001) as determined using the Kruskal–Wallis test followed by Dunn's Multiple Comparison test. None of the cytokines tested were detected in splenocyte populations stimulated with medium alone. All cytokines were detected in splenocytes from immunised mice upon stimulation with ConA (data not shown).

To analyse the T_H_ type of immune response induced in the different groups of immunised mice, we evaluated the cytokines secreted in cell culture supernatants derived from splenocytes after *in vitro* restimulation with purified Ag85B–ESAT6 protein. CBA was used to determine the secreted levels of the cytokines: IFN-γ, IL-4, IL-6, IL-2 and IL-5. Fig. 6A illustrates the mean concentrations (pg/mL) of the various cytokines secreted from spleen cells for each group on day 50 (prior to boost) and day 57 (post-boost). We observed significant levels (*p* < 0.05) of all cytokines in those mice immunised with SL3261mycolacZ alone as well as those mice also receiving i.n. boosts (either LTK63 or LTK63 plus Ag85B–ESAT6), with the exception of IL-5 levels which were not significantly different (*p* > 0.05) in SL3261mycolacZ-primed and adjuvant and Ag85B–ESAT6 boosted animals, when compared to negative controls. Indeed, levels of IFN-γ, IL-2 and IL-6 in animals boosted with adjuvant and Ag85B–ESAT6 was significantly higher when compared to those just immunised with SL3261mycolacZ alone (*p* < 0.01 compared to *p* < 0.05) (Fig. 6A). Mice immunised with SL3261 parental strain plus or minus a boost with LTK63 did not have significant levels (*p* > 0.05) of any tested cytokines on days 50 and 57 (Fig. 6A). However, mice immunised with SL3261 and boosted with LTK63 and Ag85B–ESAT6 were found to have significant levels (*p* < 0.001) of IL-4, IL-5 and IL-6, but importantly not (*p* > 0.05) IFN-γ (Fig. 6A). IFN-γ and IL-5 levels significantly (*p* < 0.05) persisted throughout the experimental period up until day 120, in those mice vaccinated with SL3261mycolacZ alone when compared to SL3261 immunised mice (Fig. 6B and data not shown). Moreover, in those mice primed with SL3261mycolacZ and boosted with LTK63 and Ag85B–ESAT6 we observed significantly higher levels (*p* < 0.05) of all cytokines tested at this late time-point (Fig. 6B and data not shown). The positive control group (i.e. those immunised with LT and Ag85B–ESAT6 were found to have modest levels of all cytokines tested at all time-points (Fig. 6 and data not shown).

### 3.4 Protective efficacy of prime-boost regimen

**Fig. 7:**
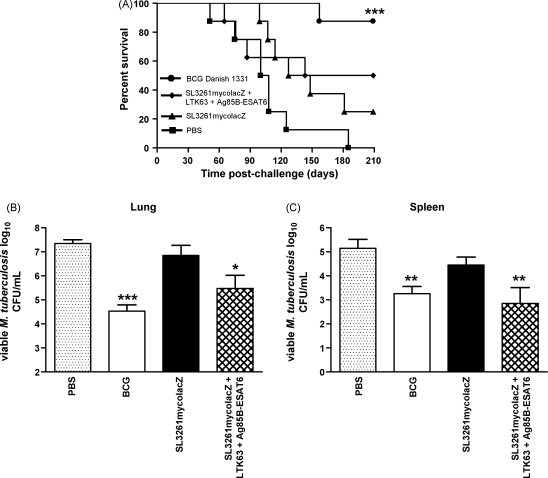
Protective responses in vaccinated animals. (A) Survival curve of guinea pigs that were aerosol-infected with *M. tuberculosis* H37Rv throughout an observation period of 210 days (*n* = 8 guinea pigs). Results are represented as Kaplan–Meier survival curves, and differences in survival were calculated by the log-rank test ***p ≤ 0.001. Influence of vaccination on bacterial load of *M. tuberculosis* in the (B) lungs and (C) spleens of guinea pigs infected by the aerosol route. Columns represent log_10_ CFU (±SD). The reduction in bacterial numbers compared to PBS controls is indicated (*p < 0.05; **p < 0.01; ***p < 0.001) as determined using the Kruskal–Wallis test followed by Dunn's Multiple Comparison test.

Challenge experiments were performed using the discriminative aerosol-infection guinea pig model to evaluate whether the strong immune responses induced in mice would confer protection against *M. tuberculosis* challenge. Ten weeks post-vaccination, guinea pigs were challenged with a low dose aerosol of *M. tuberculosis* and survival was monitored over the following 30 weeks. The experiment was terminated 30 weeks post-challenge when 100% of animals in the PBS group had reached the humane weight loss end-point (Fig. 7A). The BCG Danish s.c. group survived significantly longer when compared to the PBS group (*p* ≤ 0.001). Both SL3261mycolacZ immunised animals with and without an Ag85B–ESAT6 in LTK63 boost displayed greater survival compared to the saline group but this difference did not reach statistical significance relative to PBS (log-rank test *p* = 0.070 [30% survivors] and 0.078 [50% survivors], respectively). However, boosting SL3261mycolacZ vaccinated animals with Ag85B–ESAT6 and LTK63 was shown to improve efficacy in terms of survival to a level that was statistically equivalent to the BCG Danish (1331) vaccine unlike the group that was not boosted (log-rank test *p* = 0.009 and 0.085, respectively). Fig. 7B shows the concentration of *M. tuberculosis* in the organs from each group either when the animals had reached their humane end-point during the study or at the termination of the experiment at 30 weeks post-challenge. The BCG Danish (1331) s.c. group showed significantly reduced bacterial load (*p* < 0.01) in the lung and spleen compared to the equivalent tissues in the PBS negative control group. Orally delivered SL3261mycolacZ immunised animals showed no significant reduction (*p* > 0.05) in bacterial load in either the lungs or spleen compared to the PBS group. However, boosting SL3261mycolacZ vaccinated animals with Ag85B–ESAT6 and LTK63 was shown to not only reduce bacterial load significantly (*p* < 0.05) in both lungs and spleen when compared to the PBS control group but this protection, in terms of reduced bacterial load, was statistically equivalent (*p* > 0.05) to the BCG Danish (1331) s.c. positive control group.

## 4 Discussion

As a delivery system, live attenuated *Salmonella* vaccines are capable of eliciting an immune response to guest antigens from bacteria, parasites and viruses [10,13,43]. As bacterial vectors, both *S.* Typhi and *S.* Typhimurium have been extensively studied in both animal models and humans [44,45]. In addition, immunisation at one mucosal inductive site (e.g., intestinal) can lead to an immune response at another, anatomically remote, mucosal effector site (e.g., lungs) due to the sub-networks that exist within the mucosal immune system [46–49]. We reasoned, therefore, that a *Salmonella*-based live vector vaccine could be an excellent candidate to prime immune responses by delivering the foreign fusion antigen, Ag85B–ESAT6, directly into antigen presenting cells (APC), while providing a strong T_H_1 cytokine milieu and other immunomodulatory signals that are important for clearance and protection against tuberculosis.

Several studies have indicated that both oral and intravenous administration of recombinant *Salmonella* strains may be efficient routes of immunisation for a vaccine against tuberculosis. Previously, Hess and colleagues used *S.* Typhimurium to express and secrete the *M. tuberculosis* protein Ag85B. They transformed SL7027 with a plasmid encoding the HlyB/HlyD/TolC export machinery (from *E. coli*) and the *ag85b* gene. Those mice orally vaccinated with the recombinant *Salmonella* strain were offered partial protection after intravenous (i.v.) challenge with *M. tuberculosis* [50]. A further study also used *S.* Typhimurium as a vector to export another *M. tuberculosis* antigen, ESAT6, via the haemolysin (HlyA) secretion system. Mice immunised intravenously with this *Salmonella* vaccine expressing ESAT6 were found to have reduced numbers of tubercle bacilli in the lungs throughout the course of *M. tuberculosis* infection [51]. However, plasmid-based systems have inherent problems with stability, and antibiotic resistance genes are discouraged for incorporation into live vectors for approval for licensed clinical use [52]. Chromosomal-dependent delivery systems do have promise as they confer stability and do not require a selectable marker for maintenance. In fact, phase I human trails using the *S.* Typhi vaccine candidate ZH9 expressing LT-B antigen from the chromosome were found to be safe and immunogenic, again highlighting the advantages of utilising this approach [13]. Here, we cloned the tuberculosis fusion antigen Ag85B–ESAT6 into the chromosomal *phoN* gene of SL3261. The *ag85B–esat6* gene is under the constitutive control of the *lacZ* promoter with strong Ag85B–ESAT6 production readily detectable under *in vitro* growth conditions. Therefore this expression system would hopefully be a more realistic basis for an efficient *Salmonella* delivery system of Ag85B–ESAT6 for use in humans based upon a *S.* Typhi construct.

Prime-boost techniques, involving heterologous vaccination regimens have shown promise for inducing both cellular and humoral immune responses [53,54]. Some recent studies have examined the administration of Ag85B–ESAT6 i.n. in conjunction with a number of different mucosal adjuvants. One such study examined the immunogenicity of Ag85B–ESAT6 in combination with the adjuvant LTK63. Here, three immunisation doses given at two weekly intervals-induced strong IFN-γ responses from T cells as well as providing significant reduction in *M. tuberculosis* CFUs in both the lungs and the spleen, comparable to that seen in the BCG controls [4]. With this research in mind we also decided to incorporate our live attenuated *Salmonella* vaccine, SL3261mycolacZ, into an oral priming, i.n. boosting vaccination regimen using purified Ag85B–ESAT6 and the adjuvant LTK63.

We observed high titres of anti-Ag85B–ESAT6 Ig in the serum of mice primed orally with SL3261mycolacZ and in animals primed and i.n. boosted with purified Ag85B–ESAT6 and the adjuvant LTK63, with prominence of the IgG2a subtype. The differential production of cytokines has important effects on the Ig isotype, and the high ratio of IgG2a to IgG1 antibodies specific for Ag85B–ESAT6 is consistent with a heightened T_H_1 response, since IFN-γ is required to stimulate IgG2a secretion and is associated with inhibition of IgG1 production [55]. Indeed, the immunisation using attenuated recombinant *Salmonella* has already been reported as inducing a T_H_1-type response preferentially [11,56]. Mice primed and then boosted with or without Ag85B–ESAT6 plus LTK63 also exhibited significantly high IgA titres in both serum and lung and nasal washes.

As shown in Fig. 6, priming mice with SL3261mycolacZ was sufficient to induce significant and long-lasting levels of all cytokines tested, i.e. IL-4, IL-2, IL-5, IL-6 and IFN-γ. For a number of cytokines, their production was further elevated after i.n. boosting with Ag85B–ESAT6 and LTK63 (i.e. IL-6, IL-2 and IFN-γ). Notably, the T cell response was dominated by IFN-γ, the importance of which for the protection against *M. tuberculosis* has been shown in several studies [57,58]. IFN-γ contributes to protective immunity against *M. tuberculosis* by activating macrophages to more effectively eliminate this pathogen [59,60]. Individuals defective in genes for IFN-γ and IFN-γ receptors are prone to serious *M. tuberculosis* infections [61]. Interestingly, those animals primed with the parental SL3261 strain, and boosted with purified protein and adjuvant, exhibited strong production of the T_H_2-associated cytokines IL-4 and IL-5, however we only observed a minor T_H_2 response in SL3261mycolacZ immunised animals. A number of studies have shown that these cytokines, if produced in excess, may result in failure to control infection resulting in widely disseminated tuberculosis [62]. The differences in cytokine profiles between mice primed with the vaccine strain compared to those immunised with the negative control strain after boosting indicates the significant T_H_1 environment the recombinant SL3261mycolacZ strain induces after administration. In comparison to the recombinant *Salmonella* vaccine candidate expressing Ag85B (*S.* Typhimurium p30), constructed by Hess and colleagues, we observed that only one oral dose of SL3261mycolacZ was enough to induce approximately four times more IFN-γ than three oral doses of *S.* Typhimurium p30.

Recent studies in mice [63,64] and humans [65,66] have identified potentially dangerous side-effects of administering antigen and adjuvant i.n. Therefore, the guinea pig studies described here include mucosal priming with *Salmonella* vaccine strain and s.c. boosting with Ag85B–ESAT6 and LTK63. After aerosol challenge with pathogenic *M. tuberculosis*, guinea pigs immunised with SL3261mycolacZ alone did not have significantly improved protection when compared to PBS controls. Only those animals primed with the *Salmonella* vaccine candidate and s.c. boosted with LTK63 and Ag85B–ESAT6 were found to have statistically equivalent protection to those animals immunised with BCG. Whereas, the mouse model has been extensively used to determine efficacy of possible human *S.* Typhi vaccine candidates, very few studies have used guinea pigs. Several studies suggest that the intracellular environment of macrophages of guinea pigs may be unfavourable for the proliferation of *S.* Typhimurium [67–73]. Rapid destruction of the vaccine vector by guinea pig macrophages and consequently reduced levels of Ag85B–ESAT6 may therefore impact on the strength of the immune response and may explain why we only observed a modest increase in protection in those animals vaccinated with SL3261mycolacZ alone. Even though the immune responses induced by oral vaccination alone did not confer significant protection, when these animals were boosted with LTK63 and Ag85B–ESAT6 we did observe significantly lower *M. tuberculosis* CFU in both the lungs and spleens of these immunised mice and a protective effect which was similar to BCG vaccinated controls. These data are encouraging for using SL3261mycolacZ, or the *S.* Typhi vector equivalent, in humans as part of a heterologous prime-boost strategy. Although, the antibiotic resistance marker present in SL3261mycolacZ would have to be removed before it was an acceptable construct to be used in human vaccination. Indeed, as BCG has been used for over six decades, and over three billion doses have been administered, further studies could also investigate the use of this novel *Salmonella* vaccine as a boost to enhance and prolong the protective immunity of BCG prime immunisation.

In conclusion, we have demonstrated for the first time that mice vaccinated with attenuated *S.* Typhimurium expressing the Ag85B–ESAT6 protein via the chromosome (SL3261mycolacZ) developed potent humoral and cellular immune responses. When these primed mice were consequently i.n. boosted with the adjuvant LTK63 and Ag85B–ESAT6, we observed a further significant increase in both antibody titres and cytokine levels. Finally, when tested for protective efficacy in the aerosol guinea pig model, priming with SL3261mycolacZ and boosting with antigen and adjuvant significantly improved protection in animals that was equivalent to levels observed in BCG vaccinated controls. The data presented here provide a foundation for future studies involving an orally administered recombinant *S.* Typhi live vector vaccine stably expressing the *M. tuberculosis* fusion antigen, Ag85B–ESAT6, as part of an oral priming, boosting vaccination regimen.

## Figures and Tables

**Table 1:** Details of primers used in study.

Target DNA	Primer name	Primer sequence (5′–3′)
*lacZ*-Ag85B-EAT6 from p2795 (primers designed to include *phoN* knock-in regions [Hensel Vector])	phoNhensF	GCTGTGGCCAGTTTGCGGGAAGACTTTCACCTTCAGTAATTAAGATACGACTCACTCACTATAGGGCG
	phoNhensR	CTGTTTATTATTGCCTGATCCGGAGTGAGTCTTTATGAAAAGTTGACCATGATTACGCCAAGC

*lacZ*-Ag85B–ESAT6 (sequencing primers for chromosomal *phoNlacZ*-Ag85B–ESAT6 insert)	phoNseqF	GGTATGGACAGACGATAATGCCAGGGCA
	phoNseqR	GAATTCATGAGAATCGGGGAAACCAAAG
	mycoseqR	CGTGTTTCGCTATTCTACGCGAACTCGGCG
